# Evaluation of diagnostic performance of H-based blocking ELISA for specific detection of peste des petits ruminants in domestic sheep, goats, cattle and camels

**DOI:** 10.1186/s12866-022-02669-w

**Published:** 2022-10-21

**Authors:** Kumela Lelisa, Tesfaye Rufael Chibssa, Fanta Desissa, Kemal Emiyu, Ayelech Muluneh, Demeke Sibhatu Lobago, Dereje Shegu Gebreweld, Kebede Debebe, Abde Aliy Mohammed

**Affiliations:** 1 Animal Health Institute, P.O Box 04, Sebeta, Oromia, Ethiopia; 2 College of Veterinary Medicine and Agriculture, Department of Microbiology, Immunology and Veterinary Public health, Addis Ababa University, P.O. Box, 34, Addis Ababa, Ethiopia

**Keywords:** Peste des petits ruminants, Ruminants, HPPR- b-ELISA, c-ELISA, VNT, Antibodies

## Abstract

**Introduction:**

Peste des petits ruminants virus (PPRV) causes a highly devastating disease of sheep and goats, peste des petits ruminants (PPR), which is targeted for global control and eradication by 2030. The serological diagnostic tool kits for accurate diagnosis of PPR have inherent strengths and weaknesses that require parallel validation and optimization across animal species. Thus, the objective of this study was to evaluate diagnostic performance of haemagglutinin based PPR blocking ELISA (HPPR- b-ELISA), that was developed by Africa Union Pan African Veterinary Vaccine Center for specific detection of anti- PPRV antibodies.

**Methods:**

In preliminarily investigation, diagnostic performance of the HPPR-b-ELISA®, commercial PPR competition ELISA (c-ELISA) and virus neutralization test (VNT) were compared for the detection of anti-PPRV antibodies in goats, sheep, cattle and camels.

**Results:**

The sensitivity and specificity of HPPR- b-ELISA® were 79.55 and 99.74%, respectively, compared to c-ELISA. The HPPR- b-ELISA® was in perfect agreement (κ = 0.86) with the c-ELISA in all sera collected from goats, sheep and cattle. There was almost perfect agreement between the species of goats (κ = 0.82) and sheep (κ = 0.98), while the agreement was substantial in cattle (κ = 0.78) and no agreement was observed in camels (κ = 0.00). Similarly, the sensitivity and specificity of the HPPR b-ELISA were 80 and 96.36%, respectively compared to VNT with almost perfect agreement in goats (κ = 0.83) and sheep (κ = 0.89), moderate in cattle (κ = 0.50) and none in camels (κ = 0.00).

**Conclusion:**

Our study revealed that HPPR- b-ELISA is a suitable and valid method that can alternatively be used for screening and monitoring of PPR in sheep, goats and cattle except for camels.

## Introduction

Peste des petits ruminants virus (PPRV) causes a highly devastating disease of sheep and goats, peste des petits ruminants (PPR), that threatens food security and the conservation of wild small ruminants [1, 2]. Current efforts are directed towards control and eradication of PPR by 2030, an initiative of the Food and Agriculture Organization of the United Nations (FAO) and World Organization of Animal Health (OIE). The serological diagnostic tests landscape for PPR diagnosis (Virus Neutralization Test (VNT), immunochromatographic lateral flow devices, blocking ELISA, pseudotype-based neutralization assays, and PPR-Luciferase Immunoprecipitation System) have inherent strengths and weaknesses that require parallel validation and development [3].

Exposure to PPRV is primarily diagnosed by several serological assays that target anti-PPRV antibodies [3]. The genome of the virus is composed of six genes in the order of 3’-N, P(C/V), M, F, H, and L-5’ [4]. The encoded proteins, which include haemagglutinin protein (H), large polymerase protein (L), fusion protein (F), matrix protein (M), and phosphoprotein (P), all contain the individual genes from which they were produced [5]. Additionally, the P gene codes for the non-structural proteins C and V [6]. Since the N gene is adjacent to a genomic promoter and the most frequently transcribed gene, the N protein is abundant in PPRV-infected cells [7]. Due to its antigenic stability and abundance, the N gene and its corresponding protein are suitable candidates for the development of serological and molecular assays [8]. Most of the neutralizing antibodies are directed against the surface glycoprotein H [9, 10]. The N and H proteins are ideal candidates in the development of diagnostic tests and vaccines. The N gene is located in the vicinity of the PPRV gene, which causes the N protein to be abundant in PPRV-infected cells.

Serological tests like blocking ELISA and competitive ELISA are the leading PPRV antibody detection methods and known for their simplicity and capacity to screen large number of samples, hence, they are suitable for sero-surveillances and sero-monitoring [11]. It is important to confirm new findings through alternative diagnostic strategies such as molecular diagnostic tests since false-negative and false-positive results might occur [12].

Currently, the only commercial test available for detection of antibodies generated against PPRV is the N-based c-ELISA (ID Screen® PPR Competition, ID Vet) [13]. Nevertheless, veterinary laboratories in developing countries cannot afford to purchase this kit. To address this challenge, an alternative diagnostic test (HPPR b-ELISA®) for the detection of antibodies produced against PPRV in sheep, goats, cattle and camels has been developed by the African Union-Pan African Veterinary Vaccine Center (AU-PANVAC) [13]. However, given the results of the current investigation, further studies are needed to evaluate the validity, specificity, and sensitivity of the HPPR b-ELISA® developed in Ethiopia. The objective of this study was to compare the diagnostic efficiency of the HPPR b-ELISA® with that of the c-ELISA and VNT for the detection of antibodies produced against PPRV in sheep, goats, cattle and camels.

## Materials and methods

### Study sites

All sera sample from goats and sheep were collected from Oromia regional State, Borena zone, Yabello district, while samples from cattle were collected from Tigray regional state around Mekele city, whilst camels’ sera were collected from Afar regional state, Awash Fentale district.

### Collection of serum

A total of 480 sera samples were collected from goats, sheep, cattle and camels during the active PPR outbreak. Approximately 5 ml blood samples were collected from the jugular vein of each animal using plain vacutainer tubes. Serum was emptied and aliquoted into 1.8 ml cryovial, kept in an icebox containing icepacks, and transported to the laboratory for sample processing and laboratory investigations and temporary stored at -20 °C until tested.

### Comparison of the serological tests

#### HPPR b-ELISA

HPPR b-ELISA is PPR test developed by AU-PANVAC at Bishoftu in Ethiopia [13]. Briefly, the kit uses micro-plates pre-coated with inactivated PPRV antigen and the reagents were equilibrated for 30 min at room temperature before use. Then, 75 µl of blocking buffer was distributed to all wells, 25 µl of blocking buffer distributed to wells A1 and A2, 25 µl of positive control to wells B1 and B2 and 25 µl of negative control to wells C1, C2, D1 and D2 and 25 µl of each serum per well was distributed to the remaining wells followed by covering and incubating the plate at 25 °C for 1 h. Following three washes using 300 µl of washing buffer and blot dry in clean paper towels, 100 µl of blocking buffer was distributed in control buffer wells A1 and A2 and 100 µl of C4F3-HRP conjugate in the remaining wells. Then, the plates were covered and incubated at room temperature for 45 min. All the wells were washed three times with 300 µl of washing buffer and 50 µl of tetra-methyl-benzidine (TMB) substrate was distributed to all wells. Then, the plates were covered and incubated in a dark room for 15 min at 37 °C. Then, 50 µl of 1 mol H_2_SO4 was distributed to all wells and read OD in wells using ELISA reader (Highland Park, LTD, USA) with an inference filter at 450 nanometers connected to a computer loaded with ELISA data information software (Gen 5.3.04) for automated reading and calculation of the percentage inhibition (PI) values. The test result is said to be positive if the PI (%) value is ≥35%.

#### ID screen PPR competition ELISA

The nucleoprotein-based c-ELISA kit was used and the test carried out according to the manufacturer’s protocol (IDvet innovative diagnostics, France) [14].

### Virus neutralization test

The VNT was golden standard test used for confirmation test for ELISA test results. The test sera were thawed and deactivated by heating at 56 °C for 30 min in a water bath. Serum samples to be tested were diluted one in five and following two-fold serially diluted with minimum essential media (100 µl/well). Then, 100 µl of PPRV (vaccine strain PPRV Nigeria 75/1 allocated at AHI laboratories) at 10^3^ TCID50/ml was distributed to all wells. The control plate containing both negative and positive control was prepared separately. The Negative control contains six wells with 200 µl cell culture medium without the virus. Positive control was arranged as six wells each for 100 TCID_50_, 10, 1 and 0.1 TCID_50/_well. Plates were then stayed in the incubator for one hour at 37 °C after which 50 µl of the suspension of Vero dog SLAM cells (4 × 10^5^ cells per ml) was distributed to all wells. The plates were put in an incubator with 5 5% CO_2_ at 37 °C. Finally, the plates were followed using an inverted type of microscope, to monitor the cytopathic effect (CPE) starting day 3 of incubation. The CPE was observed when the serum was negative, in contrary, no CPE was observed if there were neutralizing antibodies in the serum against PPRV. Serum was considered positive for PPRV antibodies if the neutralizing dilution was greater than or equal to 1:10 [15, 16].

### Data management and analysis

The data from serological tests was entered into Microsoft Excel® 2013, filtered and coded and analyzed using a statistical package of STATA version 12.0. The agreement between the tests was determined using Cohen’s kappa statistics. The kappa value was interpreted as follows: Kappa value (κ) ≤ 0 as indicated no agreement, 0.01–0.20 as none to slight, 0.21–0.40 as fair, 0.41–0.60 as moderate, 0.61–0.80 as substantial, and 0.81-1.00 as almost perfect agreement [17, 18]. A standard formula recommended by Munro [19] was used to calculate the diagnostic sensitivity and specificity of the results of HPPR b-ELISA® in comparison to ID Screen® PPR c-ELISA and VNT kits. The sensitivity was calculated by the formula: Sensitivity = TP/ (TP + FN) ×100, where TP = True positive, FN = False Negative. The diagnostic specificity was calculated by the formula: Specificity = TN/ (TN + FP) ×100 and expressed as percentage where TN = True negative, FP = False positive.

## Results

The efficacy of the HPPR b-ELISA was compared with that of the ID Screen® PPR c-ELISA kit by employing of 120 goats’ sera (Table 1). The diagnostic sensitivity and specificity of HPPR b-ELISA® were 83.33 and 99.12%, respectively when compared with ID Screen® PPR c-ELISA in goats.

**Table 1:** Comparison HPPR b-ELISA and PPR c-ELISA for the detection of antibodies directed against PPRV in goats’ sera

HPPR b-ELISA	PPR c-ELISA		
	Negative	Positive	Total
**Negative**	113 (94.17%)	1 (0.83%)	114 (95.00%)
**Positive**	1 (0.83%)	5 (4.17%)	6 (5.00%)
**Total**	114 (95.00)	6 (5.00%)	120

Agreement = 98.33% Expected agreement = 90.50% κ = 0.82 SE = 0.09 P = 0.00

The HPPR b-ELISA® was found to detect 31 (25.83%) anti-PPRV antibodies, while ID Screen® PPR c-ELISA detected anti-PPRV antibodies in 32 (26.67%) in sheep sera (Table 2).

The diagnostic sensitivity and specificity of HPPR b-ELISA® relative to ID Screen® PPR c-ELISA were 96.88 and 100%, respectively.

**Table 2:** Comparison of diagnostic performance HPPR b-ELISA® with that of ID Screen® PPR c-ELISA in detecting anti-PPRV antibodies in sheep

HPPR® b-ELISA	ID Screen® PPR c-ELISA		
	Negative	Positive	Total
**Negative**	88 (73.33%)	1 (0.83%)	89 (74.17%)
**Positive**	0 (0.00%)	31 (25.83%)	31 (25.83%)
**Total**	88 (73.33%)	32 (26.67%)	120

Agreement = 99.17% Expected agreement = 61.28% κ = 0.98 SE = 0.09 P = 0.00

The HPPR b-ELISA® was found to detect 34 (28.33%) anti-PPRV antibodies, while ID Screen® PPR c-ELISA detected 46 (38.33%) antibodies directed against PPRV in cattle sera (Table 3).

The relative sensitivity and specificity of HPPR b-ELISA® in comparison with that of ID Screen® PPR c-ELISA in cattle were 73.91 and 100.00%, respectively.

**Table 3:** Comparison of HPPR b-ELISA® and ID Screen® PPR c-ELISA in detecting anti-PPRV antibodies in cattle sera

HPPR® b-ELISA	ID Screen® PPR c-ELISA		
	Negative	Positive	Total
**Negative**	74 (61.67%)	12 (10.00%)	86 (71.67%)
**Positive**	0 (0.00%)	34 (28.33%)	34 (28.33%)
**Total**	74 (61.67%)	46 (38.33%)	120 (100.00%)

Agreement = 90.00% Expected agreement = 55.06% κ = 0.78

Four (3.33%) samples that showed negative result with HPPR b-ELISA® were shown positive with ID Screen® PPR c-ELISA, whereas samples that were negative by ID Screen® PPR c-ELISA were not positive with HPPR® b-ELISA (Table 4).

The diagnostic specificity of HPPR b-ELISA® in relation to ID Screen® PPR c-ELISA was 100.00%, while that of ID Screen® PPR c-ELISA compared to HPPR b-ELISA® was 96.67%, in camels.

**Table 4:** Comparison of HPPR b-ELISA® and ID Screen® PPR c-ELISA in the detection of antibodies directed against PPRV in camels’ sera

HPPR b-ELISA®	ID Screen® PPR c-ELISA		
	Negative	Positive	Total
**Negative**	116 (96.67%)	4 (3.33%)	120 (100.00%)
**Positive**	0 (0.00%)	0 (0.00%)	0 (0.00%)
**Total**	116 (96.67%)	4 (3.33%)	120

Agreement = 96.67% Expected agreement = 96.67% κ = 0.00 SE = 0.00 P = 0.50

### Validation of HPPR b-ELISA® and ID Screen® PPR c-ELISA results with VNT

A total of 80 sera samples of all species were selected for testing. The samples that were positive with either HPPR b-ELISA® (N = 1) or ID Screen® PPR c-ELISA (N = 15), negative (N = 43), and positive (N = 21) with both tests were used to validate the results of the two ELISA tests with VNT. Compared with VNT, the HPPR b-ELISA® had a sensitivity and specificity of 80.00 and 96.36%, respectively (κ = 0.78). The agreement between HPPR b-ELISA® and VNT was almost perfect in goats (κ = 0.83) and sheep (κ = 0.89), moderate in cattle (κ = 0.50), and no agreement in camels (κ = 0.00). The relative sensitivity and specificity of the HPPR b-ELISA® test compared to VNT in each species of animals is illustrated in Table 5.

**Table 5:** The diagnostic sensitivity and specificity of HPPR b-ELISA® test relative to VNT in ruminants and camels

Parameters		Species of animals			
	Goats	Sheep	Cattle	Camels	Total
**Sensitivity**	100.00%	100.00%	50.00%	--------	80.00%
**Specificity**	94.12%	87.50%	100.00%	100.00%	96.36%

The ID Screen® PPR c-ELISA had 92.00% diagnostic sensitivity and 76.36% specificity when compared with the gold standard test with a substantial agreement (κ = 0.61). The agreement between ID Screen® PPR c-ELISA and VNT was substantial in goats (κ = 0.69) and sheep (κ = 0.78), fair in cattle (κ = 0.30), and no agreement in camels (κ = 0.00). The relative sensitivity of the ID Screen® PPR c-ELISA test compared to VNT in each species of animals is presented in Table 6.

**Table 6:** The diagnostic sensitivity and specificity of ID Screen® PPR c-ELISA test with that of VNT in ruminants and camels

Parameters			Species of animals			
	Goats	Sheep		Cattle	Camels	Total
**Sensitivity**	100%	100%		80.00%	--------	92.00%
**Specificity**	88.24%	75.00%		50.00%	80.00%	76.36%

## Discussion

In the present study, we compared the diagnostic performance of HPPR b-ELISA® test with the commercial ID Screen**®** PPR c-ELISA and validate the results of the tests against VNT for specific detection of antibodies directed against PPRV in Goats, sheep, cattle and camel field samples. The performance of the diagnostic sensitivity and specificity of HPPR b-ELISA performance was found comparable to the ID Screen**®** PPR c-ELISA.

With the 480 sera samples collected from goats, sheep, cattle and camels from different parts of Ethiopia, HPPR b-ELISA detected PPRV antibodies in agreement to the ID Screen® PPR c-ELISA sera samples of ruminants and camels. The diagnostic sensitivity and specificity of HPPR b-ELISA® test relative to ID Screen® PPR c-ELISA were 83.33 and 99.12%, in goats, 96.88 and 100% in sheep, 73.91 and100% in cattle, 0.00% and 100% in camels, respectively. The HPPR b-ELISA® and ID Screen® PPR c-ELISA showed a good correlation(K = 0.86) which is in agreement with McHugh [17] who indicated that kappa value greater than 0.6 shows adequate agreement between raters.

More specifically, the agreement between the two tests in detecting antibodies against PPRV was almost perfect in goats (κ = 0.82) and sheep (κ = 0.98) and substantial in cattle (κ = 0.78) indicating that the two tests were in agreement in detecting anti-PPRV antibodies in ruminants’ sera. More importantly, the finding demonstrated that the HPPR b-ELISA® test can be used as an alternative to ID Screen® PPR c-ELISA in detecting anti-PPRV antibodies in small ruminants and cattle. Mapaco et al.[20] used ID Screen® PPR c-ELISA as a screening test, followed by HPPR b-ELISA® as a confirmatory test to detect anti-PPRV antibody in Mozambique.

In contrast to other species, HPPR b-ELISA® and ID Screen® PPR c-ELISA had no agreement in detecting anti-PPRV antibodies in camels’ sera (κ = 0.00). Further investigations are needed why the two tests do not agree in detecting antibodies against PPRV in camels’ sera and weather a new protocol is needed in either or both tests to use them in detecting anti-PPRV antibodies in camels’ sera. For instance, previous studies revealed that the haemagglutinin protein (H)-based competitive enzyme-linked immunosorbent assay (H c-ELISA) has a lower sensitivity in cattle compared to domestic sheep and goats [21–23]. The differences between PPRV H c-ELISA and neutralisation tests in buffalo sera have also been reported, indicating that differential antiviral immune responses among host species may affect the serology and interpretation of results Jones, [24].

The study showed that HPPR b-ELISA® was slightly less sensitive than ID Screen® PPR c-ELISA which might be related to the fact that the N-protein is very important protein produced during virus replication that induce a high level of antibodies in the beginning of morbilliviruses infections or vaccination [25]. Antibodies against N-protein are easily perceptible and might contribute to the slight contrast in sensitivity compared to the HPPR® b-ELISA. The H-protein produce most of the neutralizing and defensive antibodies when susceptible animals are infected or vaccinated with morbilliviruses [26]. Nevertheless, anti-H-antibodies are also produced at a level similar to that of anti-N antibodies, and the anti-H-antibodies could be detected beginning at 7 days post-infection or vaccination. Antibodies against the H-protein are at the high level at 21 days post-infection or vaccination [27].

The sensitivity of HPPR b-ELISA® and ID Screen**®** PPR c-ELISA were 80.00 and 92.00%, respectively relative to the VNT. The HPPR b-ELISA® test had higher specificity (96.36%) than the ID Screen® PPR c-ELISA (76.36%) compared to the VNT. Both ELISA tests had excellent sensitivity (100.00%) in goats and sheep compared to the VNT. The HPPR b-ELISA® test had higher specificity (94.12%) than the ID Screen® PPR ELISA (88.24%) in compared with the gold standard test in goats. Jacobson [28] described that a diagnostic test is efficacious when diagnostic sensitivity and specificity are around 90% and above compared to the gold standard test.

Similarly, both ELISA tests had acceptable specificity, which was 75.00 and 87.50% for the ID Screen® PPR c-ELISA and HPPR® b-ELISA, respectively in sheep. The ID Screen® PPR c-ELISA and HPPR b-ELISA® had a sensitivity of 80.00 and 50.00%, respectively, while the diagnostic specificity of ID Screen® PPR c-ELISA and HPPR b-ELISA® was 50 and 100%, respectively with a fair agreement for ID Screen® PPR c-ELISA and moderate agreement for HPPR b-ELISA® in cattle. The relative sensitivity and specificity of ID Screen® PPR c-ELISA in reference laboratories were 94.5 and 99.4%, respectively [14]. Saliki et al. [29] found that the blocking ELISA using two neutralizing monoclonal antibodies had 98.9% specificity and 90.4% sensitivity in comparison to the VNT.

In camels, four sera were found to be positive using ID Screen® PPR c-ELISA, while all sera were negative using both VNT and HPPR b-ELISA®. This might indicate the ID Screen® PPR c-ELISA shows false positive in detecting antibodies against PPRV in camels. VNT negative sera that were found positive with ID Screen® PPR c-ELISA and HPPR b-ELISA® might be due to the reactivity with related antibodies produced by other morbilliviruses [30]. In fact, ruminants and camels on farms are often in contact with dogs and could share canine distemper virus infection and undergo sero-conversion. However, this assumption needs to be confirmed by VNT for specific detection of antibodies against other morbilliviruses including canine distemper virus [31].

In conclusion, both the HPPR b-ELISA® and commercial ID Screen® PPR c-ELISA tests were efficient serological methods relative to the gold standard test for the specific detection of antibodies against PPRV in goats, sheep and cattle sera. In these animal species, the HPPR b-ELISA® can be used as an alternative test for PPR screening and monitoring. Moving forward, a clear protocols and guidelines for the interpretation of PPR serological test results in a typical and wildlife host species need to be established. Parallel and replicated testing of samples with several diagnostic methods will contribute to our understanding of the respective performance and accuracy of each diagnostic test. This will help to select fit-for- purpose serology use in various epidemiological situations that may arise during and after Global PPR Control and eradication Programme.

## Data Availability

The datasets supporting the conclusions of this research article are available upon request to the corresponding author.
